# Viruses in chronic rhinosinusitis: a systematic review

**DOI:** 10.3389/falgy.2023.1237068

**Published:** 2023-12-05

**Authors:** Nitish Kumar, Tripti Brar, Hirohito Kita, Lisa A. Marks, Amar Miglani, Michael J. Marino, Devyani Lal

**Affiliations:** ^1^ Department of Otorhinolaryngology-Head & Neck Surgery, Mayo Clinic in Arizona, Phoenix, AZ, United States; ^2^ Department of Immunology and Medicine, Mayo Clinic in Arizona, Scottsdale, AZ, United States; ^3^ Library Services, Mayo Clinic Libraries-Arizona, Scottsdale, AZ, United States

**Keywords:** sinusitis, respiratory virus, chronic rhinosinusitis, viral infection, exacerbation, inflammation

## Abstract

**Background:**

Unlike acute rhinosinusitis (ARS) which is mostly viral in etiology, the role of viruses in chronic rhinosinusitis (CRS) remains unclear. Viruses may play a role in initiation, exacerbations or perpetuate chronic inflammatory responses in the sinonasal mucosa. Research needs to characterize whether viruses are part of the normal sinonasal microbiome, colonizers or pathogenic.

**Methods:**

Systematic review of the English literature was conducted. Following databases were searched with an initial search conducted in November 2021 and then updated through June 2023: Ovid Medline (1946 to present), Ovid Embase (1988 to present), Scopus (2004 to present) and Web of Science (1975 to present). MeSH (Medical Subject Headings) terms included: viruses, virus diseases, sinusitis, and rhinovirus. Keywords: virus, viral infection*, sinusitis, rhinovirus, chronic rhinosinusitis, CRS, respiratory virus, respiratory infection*, and exacerbat*. A supplementary search was conducted through September 2023: Ovid Medline (1946 to present), Epub Ahead of Print, In-Process & Other Non-Indexed Citations and Ovid MEDLINE(R) Daily. Keywords used were: virus, viral infection*, sinusitis, chronic rhinosinusitis, CRS, respiratory virus, respiratory infection*, and exacerbat*.

**Results:**

Thirty studies on viruses in CRS met inclusion criteria for full review. These included 17 studies on prevalence of virus in CRS, 5 examining probable causes of host susceptibility to viral infections in CRS, and 8 studies examining pathological pathways in viral association of CRS. The prevalence of viruses in nasal specimens of CRS subjects was higher as compared to controls in most studies, though a few studies showed otherwise. Rhinovirus was the most common virus detected. Studies showed that viruses may be associated with persistent hyper-responsiveness in the sinonasal mucosa, susceptibility to bacterial infections, upregulation of genes involved in the immune response and airway remodeling as well as CRS exacerbations. Presence of viruses was also associated with worse symptom severity scores in CRS subjects.

**Conclusion:**

Most data show higher presence of viruses in nasal and serum samples of CRS subjects as compared to controls but their exact role in CRS pathophysiology in unclear. Large studies with longitudinal sampling at all disease phases (i.e., prior to disease initiation, during disease initiation, during disease persistence, and during exacerbations) using standardized sampling techniques are needed to definitively elucidate the role of virus in CRS.

## 1. Introduction

Chronic Rhinosinusitis (CRS) is a significant public health problem afflicting 5%–12% of the global population (1). Historically, CRS was assumed to be an “infection”, but contemporary studies have moved away from this dogma. A complex interplay between host and environmental factors likely results in chronic inflammation of the sinonasal mucosa (2). The initial trigger has been hypothesized to be disruption of the sinonasal mucosal barrier by infection (bacteria, fungi, viruses), mechanical trauma, allergies, etc. The initial insult triggers a cascade of immunological responses that get dysregulated, ultimately resulting in a chronically inflamed sinonasal epithelium that is independent of the cessation of the initial insult.

Microbiome dysbiosis has been characterized as a hallmark in CRS, with CRS patients demonstrated to have microbial community collapse and loss of diversity compared to healthy controls (3, 4). In health, the normal sinonasal mucosa acts as an immuno-mechanical barrier against pathogens. In targeting pathogens, the sinonasal mucosa deploys Type 1 immune responses against intracellular pathogens, most commonly against viruses. Type 1 inflammation is associated with IL (interleukin)-2, IFN (interferon)-gamma, with canonical effector cells being M1 macrophages, Natural killer (NK) cells, CD8+ T cells, Th1 cells and ILC (Innate Lymphoid Cell) 1 cells. Type 2 immune response is directed against large extracellular pathogens such as parasites, and associated with IL-4, IL-5, IL-13, IL-25, IL-33 and IgE with effector cells that include M2 macrophages, eosinophils, Th2 cells, ILC2 cells and mast cells. Type 3 immune responses are directed towards extracellular bacteria and fungi, and is associated with cytokines IL-17A, IL-17F and IL-22, with the effector cells being neutrophils, Th17 cells and ILC3 (5). In the United States and the Western hemisphere, CRS with nasal polyps (CRSwNP) is traditionally characterized by a predominantly Type 2 inflammatory profile response, whereas CRS sine (without) nasal polyps (CRSsNP) has been classically associated with type 1 and type 3 responses, however there is heterogeneity within endotype and phenotype correlation (6). In the United States, data shows that a significant number of CRSsNP may have Type-2 features (7, 8).

Given the inflammatory profile in CRS, microbes may have a significant role in causing initial immuno-mechanical insult to the respiratory and sinonasal lining that results in a chronic inflammatory condition. Unlike acute rhinosinusitis (ARS) which is mostly viral in etiology (9–11) and self-limiting, chronic rhinosinusitis is characterized by unremitting inflammation of the sinonasal mucosa. In addition to disease initiation, virus may play a role in the acute exacerbations of the chronically inflamed state in addition to potentially perpetuating chronic inflammatory responses in the sinonasal mucosa. Though certain respiratory viruses such as rhinovirus have been found more commonly in nasal brushings/tissue samples in patients with CRS vs. healthy controls (12–15), the role of viruses in CRS remains unclear (16) and further research is necessary to elucidate and characterize viruses in the sinonasal cavity as part of the normal microbiome, colonizers or pathogens.

**Figure 1:**
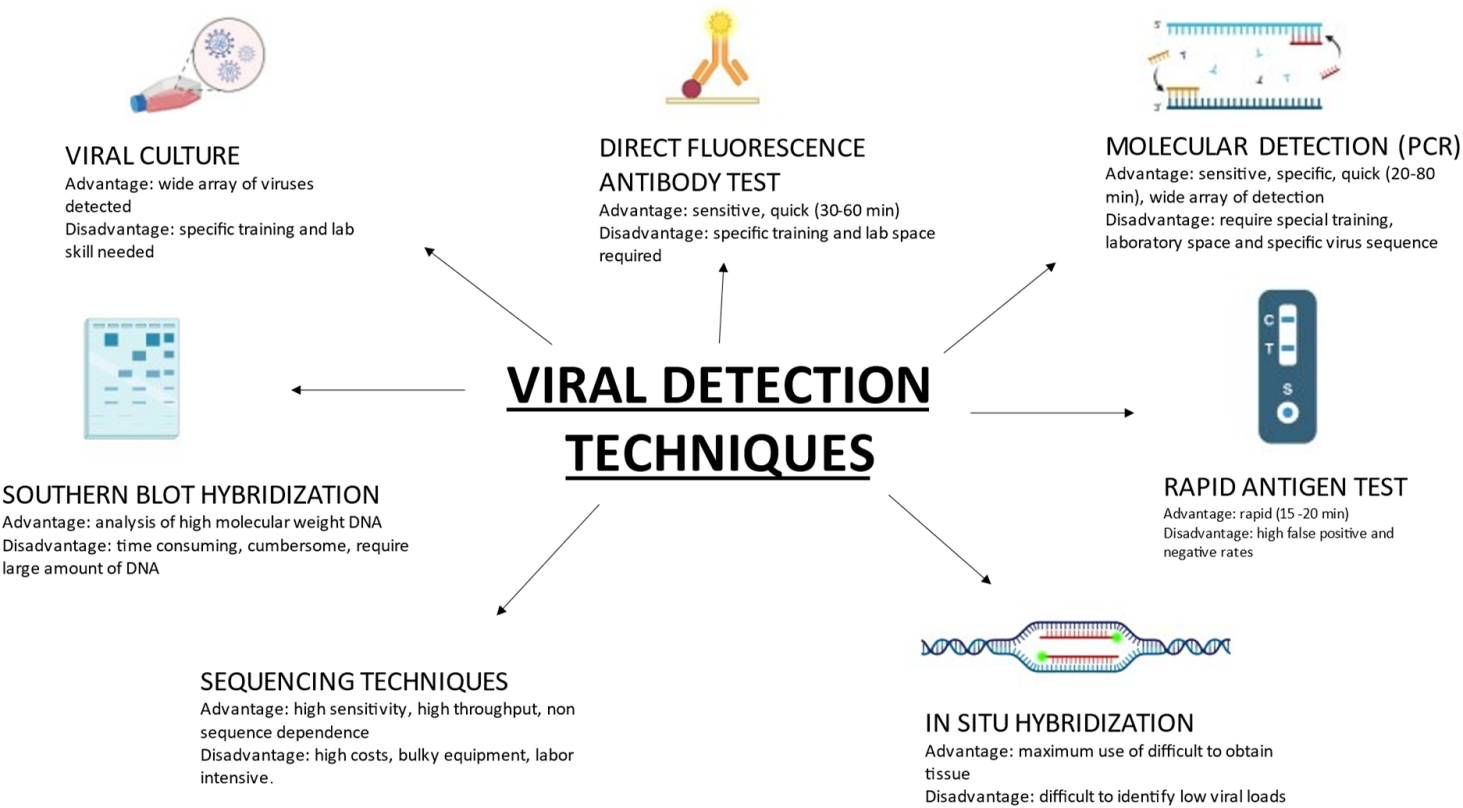
Contemporary viral detection techniques in common use, with specific advantages and disadvantages of each.

Until a few years ago, viral detection was time consuming as it was culture based; however, with the use of molecular—Polymerase chain reaction (PCR) based techniques, detection is more sensitive, samples can be rapidly analyzed, and new serotypes of viruses identified. Presented in Figure 1 are viral detection techniques that are currently in use, with their advantages and disadvantages.

This systematic review scrutinizes the contemporary literature for studies on viruses in CRS initiation, exacerbation and persistence.

## 2. Materials and methods

### 2.1. Study design

A systematic review of the English literature was conducted. The following databases were searched initially in November 2021 then updated though June 2023: Ovid Medline (1946 to present), Ovid Embase (1988 to present), Scopus (2004 to present) and Web of Science (1975 to present). A combination of MeSH (Medical Subject Headings) and keywords were used. The MeSH terms included: viruses, virus diseases, sinusitis, and rhinovirus. Keywords used: virus, viral infection*, sinusitis, rhinovirus, chronic rhinosinusitis, CRS, respiratory virus, respiratory infection*, and exacerbat*. The MeSH terms and counterpart keywords were combined using the Boolean operator “OR” then OR'd concepts were combined using the Boolean operator “AND”. Finally, the MeSH term and keyword of asthma was eliminated from the search strategy using the Boolean operator of “NOT”. (* indicates truncation of word or phrase).

The following databases were searched again through September 2023 to supplement the previously searched data: Ovid Medline (1946 to present), Epub Ahead of Print, In-Process & Other Non-Indexed Citations and Ovid MEDLINE(R) Daily. A combination of MeSH (Medical Subject Headings) and keywords were used. The MeSH terms included: viruses, virus diseases, sinusitis, and rhinovirus. Keywords used: virus, viral infection*, sinusitis, chronic rhinosinusitis, CRS, respiratory virus, respiratory infection*, and exacerbat*. The MeSH terms and counterpart keywords were combined using the Boolean operator “OR” then OR' concepts were combined using the Boolean operator “AND”. Finally, the MeSH term and keyword of asthma was eliminated from the search strategy using the Boolean operator of “NOT”.

### 2.2. Eligibility criteria

EndNote X9 software (Clarivate Analytics, Philadelphia, PA) was used to compile the studies. A preliminary screen was conducted by three reviewers (N.K., T.B., D.L) in which the titles and abstracts were reviewed. Criteria for inclusion was any mention of virus and sinusitis, nasal epithelial cells, and upper airway. Exclusion criteria were acute sinusitis, cystic fibrosis, chronic obstructive pulmonary disease, immunocompromised or any other non-CRS condition. Additional exclusion criteria were review articles, book chapters and abstracts without full text. A secondary screen was then conducted by all the reviewers in which full text articles were evaluated by each reviewer.

### 2.3. Data extraction

Three authors (N.K., T.B., D.L) evaluated each full text article. The authors then looked at the bibliography and found additional articles that offered insight into the role of viruses in CRS and included them in the review and discussion. Data that were extracted included subjects (animal/human), number of subjects, brief study description (including site of sampling), viruses studied and findings of the study.

## 3. Results

**Figure 2:**
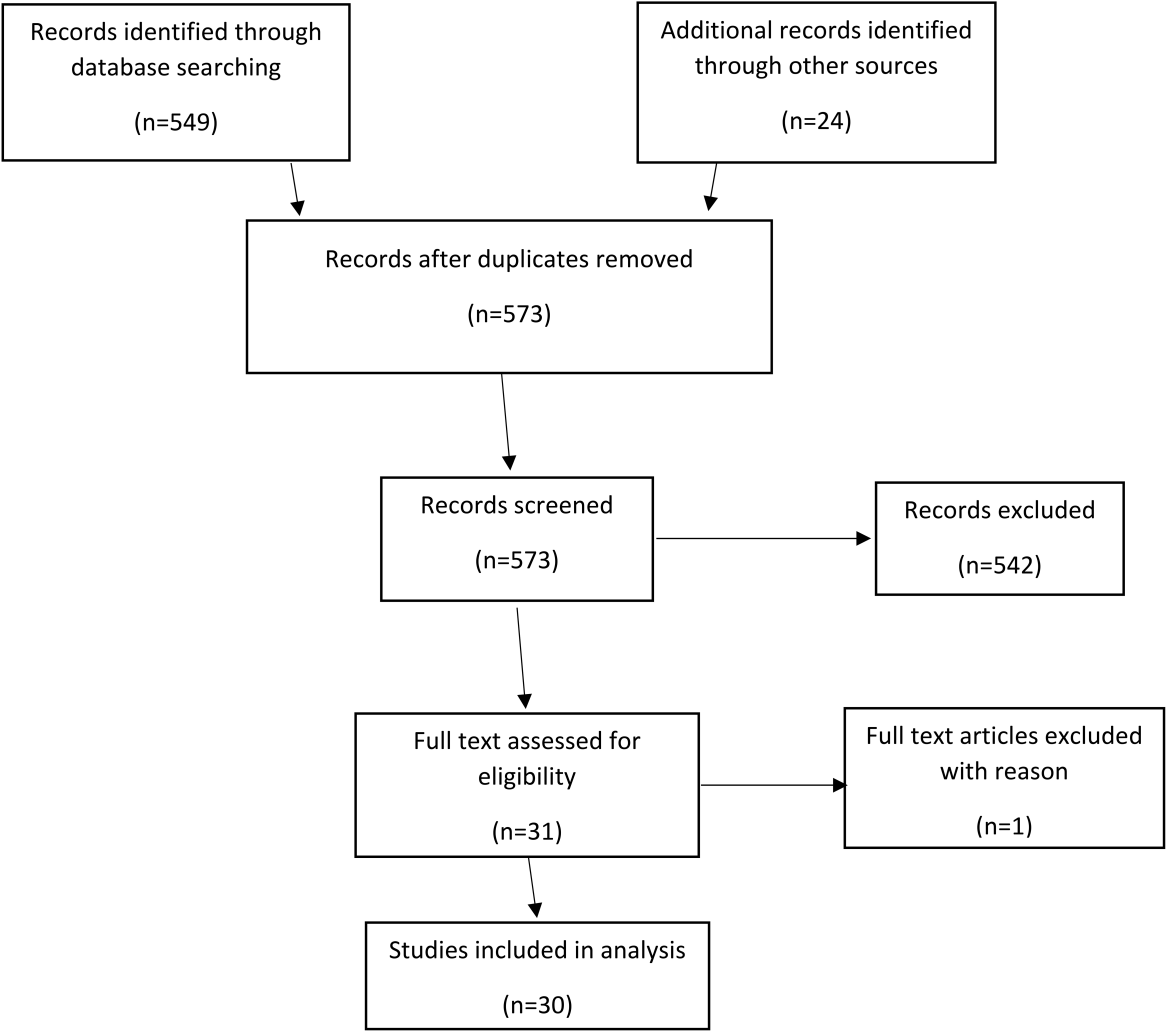
PRISMA diagram showing methodology of systematic review. (PRISMA, preferred reporting items for systematic reviews and meta-analyses).

The results of the literature search and subsequent screenings are shown in the PRISMA (Preferred Reporting Items for Systematic Reviews and Meta-Analyses) diagram in Figure 2. The search resulted in 549 (517 + 32) studies. After review of the abstracts, 6 (2 + 4) articles were included for full review of the manuscript. Search was conducted multiple times with change in keywords but did not reveal any significant difference in the quality of results. This could indicate a problem in indexing of the articles. Reference lists of the shortlisted articles were studied to identify missing articles, not identified in literature search, satisfying the inclusion criteria. 24 articles were identified during this manual search and after full review, total 30 articles included in the study.

Some studies further subclassified CRS based on phenotype (CRSwNP and CRSsNP) or endotype (eosinophilic CRSwNP and noneosinophilic CRSwNP). The number of subjects within subgroups ranged from as 2 to 133. The studies were further categorized into studies regarding the prevalence of virus in CRS subjects (17), studies investigating the causes of host susceptibility to viral infections in CRS (5) and those investigating role of virus in CRS immunopathogenesis (8).

### 3.1. Samples studied

The samples studied in human studies were variable and included nasal mucosa, uncinate tissue, brush swabs from middle meatus or ethmoidal sinus, inferior turbinate tissue and nasal lavage fluid. One study investigated serum antibody levels to 47 viral antigens (17).

### 3.2. Prevalence of virus in CRS patients

Seventeen studies investigated the prevalence of various airway viruses in sinonasal samples of CRS subjects. Some studies investigated an array of respiratory viruses (Table 1A) whereas others studied 1 or 2 specific viruses (Table 1B). Polymerase Chain Reaction (PCR) testing was the most common mechanism used for study, followed by Enzyme Linked Immuno Sorbent Assay (ELISA). One study did not sample the sinonasal mucosa at all but looked at serum antibody profiles to 47 viral antigens (17). The viruses studied included subtypes of Rhinovirus (RV), Influenza virus, Parainfluenza virus (PIV), respiratory syncytial virus (RSV), adenovirus (AdV), coronavirus (CoV), Bocavirus (BoV), human metapneumovirus (HMPV), human papilloma virus (HPV), herpes simplex virus (HSV-1/-2), varicella zoster virus (VZV), Epstein Barr virus (EBV), cytomegalovirus (CMV), and human herpes virus (HHV-6/-7). Eleven studies showed a higher prevalence of one or more viruses in patients of CRS vs. controls (12–15, 17, 19–21, 25, 26, 29). Most commonly identified viruses include HRV (13–15, 17, 19, 26), EBV (20, 25, 29), CoV (12, 19), HMPV (21). Six showed either no viral detection, or no significant difference in presence of viruses between CRS subjects and controls (18, 22–24, 27, 28).

**Table 1A:** Studies showing prevalence of common airway viruses in CRS.

Number	Author/year	Study description	Human/animal; number of subjects	Viruses studied	Findings
1.	Lal et al. (17)	Serum samples were examined for sero reactive microbial proteins using a CRS focussed NAPPA array with 1,557 microbial proteins for 47 bacteria and viruses each.	Human, CRS subjects-39 Controls-79	47 bacteria and 47 viruses	Significantly increased anti-microbial proteins in serum samples of CRS patients as compared to controls. Sero reactivity of S. aureus, HMPV, HHV-5 and influenza A (H3N2, H1N1) significantly increased. Increased sero reactivity for S. aureus, influenza A and RV-B14 in nasal polyposis; and for HHV-1 and vaccinia virus in without polyposis CRS.
2.	Hwang et al. (18)	Mucosal scraping from MM was studied for presence of viruses and sinonasal epithelial cells harvested from ethmoid sinus tissue in CRSwNP, CRSsNP and controls was investigated for the expression of Toll-like receptor (TLR) 3, TLR7, and IFN and IFN stimulated genes (ISGs)	Human; Eosinophilic CRSwNP- 35, Noneosinophilic CRSwNP- 38, CRSsNP- 36, Controls-21	16 respiratory tract viruses, including AdV, RV, and CoV	Respiratory virus detection rate was not significantly different among the groups. Decreased levels of IFN and ISGs in patients with CRS was observed, possibly showing impairment of antiviral response in CRS patients.
3.	Goggin et al. (19)	Studied sinonasal virome using cytobrush samples from sinonasal passages of patients with CRS vs. controls. Also studied the relation of viral presence to disease phenotype, and association with disease severity.	Human; CRSwNP- 84, CRSsNP- 133, Controls- 71	AdV, HBoV, CoV, enterovirus, influenza, HMPV, PIV 1–4, RSV and RV	Of the total 288 patients, 45 were positive for presence of viruses. Rate of viral detection was significantly higher in patients of CRSsNP. Virus positivity was associated with disease severity in CRSsNP but not in CRSwNP.
4.	Goggin et al. (20)	Sinonasal mucosa sampled using cytobrush at both MM and inferior meatus on right and left side to look for presence of respiratory viruses in CRS patients and controls.	Human; CRSwNP- 8, CRSsNP- 7, Controls- 9	RV, influenza A–C, PIV 1–4, RSV A and B, CoV (HKU-1, OC43, NL63, and 229E), enterovirus, hMPV, AdV, HBoV, polyomaviruses, WUPyV and KIPyV, EBV, CMV, HHV6, HSV 1 and 2, and VZV	75% of subjects were positive for at least one virus from at least one site. However, discord in viral species between sites.
5.	Lima et al. (21)	To detect respiratory virus in secretions and tissue samples (tissues obtained from the paranasal sinus mucosa, MTM, NP, and saline nasal wash) from patients with CRS and study its seasonality.	Human; CRS- 100	HRV, human enterovirus, RSV A and B, HMPV A and B, human influenza virus A and B, PIV-1 and PIV-3, CoVs (OC43 229E), AdV, and HBoV	Viruses detected in 54% of subjects. Pattern of respiratory virus seasonality in CRS patients coincided with seasonality of viruses, possibly implying asymptomatic viral infections.
6.	Rowan et al. (12)	To detect respiratory viruses in CRS and controls and correlate clinical and radiographic measures of CRS with viral presence. Brush swab from MM or ethmoid sinus was taken.	Human; CRSwNP-13, CRSsNP-8, controls-14	Influenza A H1, Influenza A H3, Influenza A H1N1 2009, Influenza B, RSV A and B, PIV 1–4, HMPV, HRV, AdV B/E and C, CoV (229E, OC43, NL63, HKU1)	Viral presence more common in CRS vs. controls. Viruses may have a role in symptom exacerbation in CRSsNP vs. CRSwNP.
7.	Liao et al. (22)	Control subjects, CRSwNP, CRSsNP patients without signs of acute viral infection were enrolled. Epithelial cells scraped from the MM were evaluated for nine common respiratory viruses.	Human; CRSwNP- 67, CRSsNP- 61, Controls- 51	Picornavirus, RSV, Influenza type A and B, PIV 1–3, CoV 229E and OC43	A high frequency of viral infection could be observed in the MM, however, no difference in frequency of viral infection in the three groups-CRSwNP, CRSsNP and controls.
8.	Costa et al. (23)	To evaluate the prevalence of certain viruses in specimens from patients with NP undergoing FESS. Samples studied were NP, turbinate mucosa, pre and postoperative turbinate scrapings.	Human; CRSwNP- 35	HHV 1–6, VZV, EBV, CMV; influenza A and B viruses, RSV A and B, AdV, HMPV, CoV 229E/NL63 and OC43, PIV 1–4, RV A/B/C, enteroviruses, and HBoV 1/2/3/4	60% of patients were positive to at least one virus. Higher EBV frequency seen in NP and HHV-6 in healthy turbinate mucosa, although not statistically significant.
9.	Cho et al. (15)	To determine prevalence of respiratory viruses in CRS patients and non-CRS controls. NLF and turbinate epithelial cells.	Human; CRS-111, Controls- 50	RV and enteroviruses, PIV 1–4, influenza viruses A and B, RSV A and B, CoV229E/NL63 and OC43, AdV, HMPV, and HBoV 1, 2, 3, and 4	Higher prevalence of respiratory virus infection in CRS patients than in controls. RV was the most prevalent virus and the only virus that had a significantly different detection rate in two groups.
10.	Wood et al. (24)	Sinus mucosa from ethmoid and sphenoid sinuses was sampled from patients with CRS and controls to look for common respiratory viruses.	Human; CRS-13, Controls- 2	PIV1, 2, and 3, RSV, HMPV, AdV, RV, CoV, HBoV, CMV and influenza A and B	No viruses detected in any of the samples.
11.	Zaravinos et al. (25)	NP and MTM and ITM were studied for presence of viruses.	Human; CRSwNP- 23, controls- 13	HPV, HSV-1/-2, VZV, EBV, CMV, and HHV-6/-7	EBV was found to be present in NP and was statistically significant. Other viruses studied here do not seem to play a significant role in polyp formation.

**Table 1B:** Studies demonstrating prevalence of particular viruses in CRS patients.

1.	Lee et al. (13)	NLF and turbinate epithelial cells to determine HRV serotypes in CRS patients and non-CRS controls.	Human; CRS-111, controls-51	HRV	Significantly high prevalence of HRV in CRS vs. controls. Higher prevalence seen in both NLF and turbinate epithelial cells. HRV-A13 was most common serotype in both CRS and controls.
2.	Abshirini et al. (26)	Conducted on patients with CRS who were candidates for FESS. Sample- Mucus from sinuses was collected.	Human; CRS- 76	RV, RSV	Approximately 33% patients had at least one virus, with RV being more common than RSV.
3.	Divekar et al. (27)	Nasal secretions studied for local and systemic immune responses associated with acute worsening of sinonasal symptoms during exacerbation in CRSwNP compared to controls. Virus detection was performed on nasal washes.	Human; CRSwNP- 9; Controls- 10 (total 22 controls and 23 CRSwNP but virus studied only in those with exacerbation)	HRV/enterovirus	No significant difference in HRV detection in CRS vs. controls during acute exacerbations. Increased levels of IL-6 in CRSwNP patients at baseline was seen. A local immune response with elevated IL-5, IL-6 and eosinophil major basic protein (MBP) in nasal secretions between CRSwNP and controls during acute exacerbation.
4.	Jang et al. (14)	NLF and turbinate epithelial cells from sinusitis patients and control subjects were evaluated.	Human; CRS-39, controls -27	Picornavirus/RV	Significant difference in RV detection in turbinate epithelial cells of CRS vs. controls. No virus detected in any group in NLF.
5.	Ramadan et al. (28)	Maxillary and ethmoid sinus tissue was used to investigate the role of viruses in CRS patients undergoing surgery.	Human; CRS- 20	AdV, RSV	20% of cases were positive for RSV, and none for AdV.
6.	Tao et al. (29)	NP tissue (preserved specimens) of patients studied for the presence of EBV.	Human; CRSwNP- 13	EBV	85% specimens tested positive for EBV, very low numbers of EBV positive cells were found in each case. Possibly a role of viral persistence.

Rowan et al. used nasal brush swab samples in 13 CRSwNP, 8 CRSsNP and 14 controls and found 24% prevalence of viruses in CRS cases, in which 50% prevalence as associated with CRSsNP and only 8% with CRSwNP; highest prevalence was found of CoV using real time polymerase chain reaction (rt-PCR) technique for viral detection (12). Lee et al. used nasal lavage samples for detection of only RV in 111 CRS patients and 51 controls and found a higher prevalence of 36% in CRS as compared to 20% in controls using rt-PCR technique. Further subtyping reveled maximum prevalence of RV-A serotype (13). Cho et al. also used nasal lavage samples along with inferior turbinate scrapings and found a higher rate of viral detection in nasal scrapings (64%) as compared to nasal lavage (50.5%). Prevalence was significantly high in cases as compared to controls and RV was the most common virus identified. They also noticed a higher rate of co-infection of viruses in cases (24.3%) as compared to controls (4.0%) (15). Jang et al. used similar technique in a smaller sample size of 39 cases and 27 controls where all nasal lavage samples were negative for viruses whereas inferior turbinate scrapings had a 21% viral detection rate in cases and none in controls (14). Lima et al. attempted to identify the seasonal variation of viral prevalence in CRS where she studied 100 CRS patients over a period of 2 years and found a viral detection rate of 54% in CRS subjects. Most common identified viruses were HMPV followed by HRV. Co-infection was found in 44% of subjects and maximum seasonal correlation was demonstrated by HRV followed by HMPV (21). Abshirini et al. did a case only study with 76 CRS cases undergoing endoscopic sinus surgery where they collected mucus specimen from ethmoidal sinus during surgery for viral detection using rt-PCR and found an overall prevalence of 32.89%, 28.94% for RV and 11.84% for RSV (26). Zaravinos et al. sampled polyp tissue for detection of viruses using rt-PCR where they found maximum prevalence of EBV followed by HPV (25). Goggin et al, in an attempt to find the most effective way of sampling to obtain highest viral yield, detected a prevalence of 75% using a cytology brush for nasal mucosal sampling and found maximum prevalence of EBV in CRS patients (20). In another study for detecting viral prevalence in 288 subjects, they found maximum viral positivity associated with CRSsNP—20.3%, compared to CRSwNP—15.4%, and controls—7%. In this study the most common identified viruses were RV and CoV. Also, to find the effect of viral association on disease severity, they found higher Lund-Mackay and Lund-Kennedy scores only in patients of CRSsNP with viral association (19). Tao et al. used southern blot hybridization, PCR and *in situ* hybridization for EBV encoded small nuclear RNA (snRNA) for detection of EBV in 13 patients of nasal polyposis and found the detection rates of 15%, 69% and 85% respectively (29).

The studies that showed no significant difference in the prevalence of viruses in CRS patient include that by Divekar et al. where they detected no difference of prevalence of RV and enterovirus (EV) between CRS patients and controls, but detected a significantly higher levels of IL-6, IL-5, VEGF (Vascular endothelial growth factor), GM-CSF (Granulocyte monocyte colony stimulating factor) and eosinophilic major basic protein (EMBP) in CRSwNP patients (27). Wood et al. used PCR for detection of common airway viruses in sinonasal mucosal samples but didn't detect any virus in the 13 subjects as well as 2 controls (24). Costa et al. used polyp tissue, turbinate mucosa and pre and post operative scrapings for detection of community acquired respiratory viruses and found no significant difference in the viral prevalence among cases and controls (23). Liao et al. and Hwang et al. also attempted to detect the viral prevalence using nasal swabs and middle meatal scrapings using rt-PCR but didn't find any significant difference in prevalence amongst cases and controls (18, 22).

### 3.3. Host susceptibility to viral infection

Five studies investigated host factors in CRS patients that might be responsible for increased susceptibility to viral infection and pathogenicity (Table 2). These studies found reduced anti-viral cytokine (IFN-γ, IL-17) levels indicating compromised antiviral defense mechanisms (34), increased permeability of the inflamed mucosa to viruses leading to increased viral invasion (34), mutations in CDHR3 viral receptor leading to increased RV-C binding (33), increased viral binding per unit area in inflamed nasal tissue in murine model (32), ephrin A1/A2 receptor mediated dysfunctional innate immune response, again indicating compromised antiviral defense mechanisms (31). Lee et al. found that in CRS subjects, air liquid interface (ALI) culture of cells obtained from ethmoidal sinus did not show any significant difference in the levels of IFN-β or IFN stimulated genes (ISGs) like viperin vs. controls after RV-16 infection, which was in contrast to findings of above mentioned studies and denies role of compromised anti-viral IFN response in virus mediated pathogenesis of CRS (30).

**Table 2:** Studies demonstrating host susceptibility.

1.	Lee et al. (30)	CRSwNP and control sinonasal tissues (uncinate process and ethmoid sinus mucosa for controls) were examined for expression of anti-viral IFN and IFN stimulated genes post RV infection.	Human; CRSwNP-59, controls- Uncinate-50 Ethmoids-15	RV	No difference in the levels of anti-viral IFN levels post infection with RV in case vs. controls, hence, not the cause of dysregulated immune response in CRS patients.
2.	Lee et al. (31)	Ethmoid sinus mucosa from controls (blowout fracture) and cases (during ESS) taken and cultured, ephA1/A2, phosphorylated ephA2 levels measured in the cell cultures, after treating with type 2 inflammatory mediators, poly(I:C), and HRV16. Using eph2 receptor blocker and siRNA ephA2 on cell cultures infected with HRV16 and measure cytokine and viral replication levels. Analysis of downstream signalling pathways.	Human Controls-25 CRSwNP-73 CRSsNP-36	RV	Raised levels of ephA1/A2 and phosphorylated ephA2 in inflamed cell cultures regardless of polyp status, in all poly(I:C), HRV 16 infected, and treated with type 2 inflammatory mediators cell cultures. Dose dependent cytokine secretion on treating with ephA2, silenced by ephA2 blocker and ephA2 siRNA. ephA2 blocking leading to reduced levels of inflammatory mediators and reduced rate of replication of HRV.
3.	Lee et al. (32)	Study of inflammatory markers and histological changes in a murine model of CRS post RV-1B infection.	Animal—mouse	RV-1B	After 48 h of RV-1B infection, there was no significant difference in levels of inflammatory markers between cases and controls, no significant histological changes were identified, but increased RV-1B infection per unit area was identified with immunofluorescence.
4.	Chang et al. (33)	Detection of rs6967330 risk allele for CDHR 3 receptor mutation in cases and controls to establish its association in etiology of CRS.	Humans Cases—	RV-C	Presence of SNP rs6967330 risk single/double allele significantly increased the odds of CRS in study population in both additive and dominant models.
5.	Lan et al. (34)	Ex-vivo model to study differences in antiviral defense in CRSwNP mucosal tissue compared to controls healthy mucosal tissue upon HSV-1 exposure.	Human	HSV-1	Significantly higher viral invasion scores at 48 and 72 h in CRSwNP mucosa compared to controls. CRSwNP mucosal tissue showed a significant deficit in IFN-γ and IL-17 release within 24 to 72 h after infection and higher pro-inflammatory cytokines in comparison to controls.

### 3.4. Role of viruses in pathophysiology of CRS

Eight studies investigated the role of viruses in persistent chronic inflammation of CRS as well as in periodic exacerbations (Table 3). Of them, 4 focused on the immuno-epithelial barrier disruption caused by viral infection as a mechanism leading to persistent inflammation and increased susceptibility to bacterial infections (35, 39, 42, 43). These studies were conducted on cultured epithelial cells and found disruption of the epithelial barrier via increased expression of Oncostatin M (OSM) post H3N2 virus infection (37), increased phosphorylation of protein kinase D (PKD) leading to destabilization of actin cytoskeleton and disruption of apical junctional complexes (AJCs) confirmed via immunofluorescence and confocal microscopy (38). Increased adherence of bacteria was found via upregulated expression of cellular adhesion molecules (CAM) in cultured epithelial cells post RV-16 infection and increased adhesion of bacteria like *S. aureus, S. pneumoniae* and *H. influenzae* compared to controls was visualized on confocal microscopy and immunofluorescence (40). Wang et al. also noticed increased mucosal invasion and *S. aureus* infection in cultured nasal epithelial cells of patient with CRSwNP as compared to controls on infection with HSV-1 (39). Higher levels of inflammatory mediators like IL-25, IL-1β, IL-10, IL-5 and tumor necrosis factor (TNF) -α after viral infection were found in human nasal epithelial cell (HNEC) culture compared to controls (34, 35). Persistent hyper responsiveness of nasal mucosa post viral infection was demonstrated in a mouse model after infection with sendai virus (SeV) where elevated levels of CD8+, CD-4+ and CD-25+ cells were found after resolution of acute phase of viral infection along with more severe symptoms on histamine challenge test (42).

**Table 3:** Studies showing pathophysiology of viral association with CRS.

1.	Hong et al. (35)	CRSwNP, CRSsNP and control sinonasal tissues (uncinate process for controls) were examined for IL-25 expression and type 2 inflammatory cytokines.	Human; CRSwNP-60, CRSsNP-25, controls- 15	Influenza, RV, RSV	Significantly elevated IL-25- (both protein and mRNA) and type 2 inflammatory cytokines were seen in CRSwNP as compared to CRSsNP and controls.
2.	Willis et al. (36)	HRV C infection identified as one with worse WURSS scores. Difference in viral replication rates, gene expression of immune signalling pathways, and levels of type 2 inflammatory mediators compared in cell cultures infected with control virus, HRV A, and HRV C.	Human, 218 subjects.	HRV A, HRV C	WURSS questionnaire collected over 2 years along with nasal swabs from 218 patients, along with detection of HRV A, HRV C revealed worse WURSS scores in females and with HRV C infection. In human MT cell cultures, infected with HRV A, HRV C, and mock virus; no difference in viral replication levels, downregulation of TGF *β*, *α*6/β4, Notch pathway in HRV A, of pro apoptotic and stress response pathways in HRV C, with upregulation of type 2 inflammatory mediators in HRV C > HRV A.
3.	Tian et al. (37)	OSM and TJs expression was measured and compared between ITM from healthy controls and NP from CRSwNP.	Human; CRS- 83, Controls- 48	Influenza virus H3N2	OSM expression in CRSwNP correlated with loss of TJs. TJ integrity was maintained in controls.
4.	Rezaee et al. (38)	In vitro study to investigate the effect of RSV on phosphorylation of PKD pathway leading to disruption of AJCs.	Human	RV	In vitro live RV induces phosphorylation of PKD leading to disruption of AJCs (tight junctions, Zonula Occludens 1, occludins) leading to decreased Transepithelial Electrical Resistance (TEER).
5.	Wang et al. (39)	ITM and NP samples cultured from healthy controls and CRS and infected with HSV-1, *S. aureus* or both.	Human; CRS- 7, Controls- 10		HSV-1 may facilitate invasion of *S. aureus* into the nasal mucosa. NP tissue was more susceptible to epithelial damage by HSV-1 compared with ITM.
6.	Wang et al. (40)	HNEC obtained from ITM of healthy subjects infected with RV *in vitro*, followed by *S. aureus, S. pneumoniae, H. influenzae.*	Human	RV	RV enhanced expression of Fn, PAF-γ, CEACAM leading to increased adherence of FITC labelled bacteria like *S. aureus, S. pneumoniae, H. influenzae* confirmed by confocal microscopy.
7.	Wang et al. (41)	In vitro study to investigate the effect of RV infection on the expression of MMPs, TIMP-1 and VEGF in NP fibroblasts derived from CRSwNP subjects.	Human	RV	RV-16 infection significantly enhanced the gene and protein expressions of MMP-2, MMP-9, and VEGF in NP fibroblasts, whereas TIMP-1 expression was not significantly affected. Possible role of viruses in CRSwNP.
8.	Klemens et al. (42)	Murine model of viral rhinosinusitis; RCT. Mice intranasally inoculated with Sendai virus (SeV) or ultraviolet (UV)-inactivated virus. On days 3 and 10 postinfection, NLF was obtained for viral culture. On days 4, 10, and 38 postinfection, sinus mucosa was analyzed for cytokines. Nasal hyperresponsiveness to histamine challenge was measured on days 8 and 36 postinoculation.	Animal (mice)	Sendai virus	Infected mice developed a significant increase in T-suppressor and T-regulatory cells even after resolution of the acute infection, which persisted for at least 38 days.

Abbreviations for viruses (in alphabetical order): AdV, adenovirus; CoV, coronavirus; CMV, cytomegalovirus; EBV, epstein barr virus; HBoV, human bocavirus; HHV, human herpes virus; HMPV, human metapneumovirus; HPV, human papilloma virus; HRV, human rhinovirus; HSV, herpes simplex virus; PIV, parainfluenza virus; RSV, respiratory syncytial virus; RV, rhinovirus; VZV, varicella zoster virus.

Other abbreviations (in alphabetical order): CARS, chronic allergic rhinosinusitis; CRS, chronic rhinosinusitis; CRSwNP, chronic rhinosinusitis with nasal polyps; CRSsNP, chronic rhinosinusitis without nasal polyps; FESS, functional endoscopic sinus surgery; hNECs, human nasal epithelial cells; IFN, interferon; IL, interleukin; ISGs, interferon stimulated genes; ITM, inferior turbinate mucosa; MM, middle meatus; MMPs, matrix metalloproteins; MTM, middle turbinate mucosa; NLF, nasal lavage fluid; NP, nasal polyp; OSM, oncostatin M; RCT, randomized controlled trial; TJ, tight junction; TIMP, tissue inhibitor of metalloproteinase; TLR, toll like receptor; VEGF, vascular endothelial growth factor.

## 4. Discussion

### 4.1. What is the role of viruses in rhinosinusitis?

Viruses have an established role in the etiopathogenesis of acute rhinosinusitis (ARS), defined as rhinosinusitis lasting less than 12 weeks (12–14). ARS is usually a self-limiting condition, but sometimes viral ARS leads to secondary bacterial infection due to epithelial changes, microbial dysbiosis, immune suppression and changes in the local environment favoring the growth of bacteria (38–40, 43, 44). Rhinovirus is the most commonly implicated virus in ARS (45, 46).

In patients with CRS, respiratory viruses are often found in nasal samples, but their role is not fully understood and yet to be firmly elucidated (13, 15, 26). Virus may cause sinonasal mucosal inflammation, disruption of the immuno-mechanical barrier at the sinonasal epithelium, increased susceptibility to bacterial adherence and resultant immuno pathogenic mechanisms at the cellular and tissue levels that result in persistent sinonasal inflammation characteristic of CRS (31, 47). Viruses may alter host gene expression, leading to altered patterns of immune response and pathogenic changes in levels of cytokines/chemokines (35, 36, 48, 49). Additionally, viral infections may lead to alteration of the normal sinonasal microbiome, which could lead to a cascade of events causing inflammation of the upper airway (47).

In vitro studies have shown that after a viral infection, host antiviral response genes may inhibit ciliogenesis and ciliary function of nasal epithelial cells, ultimately leading to chronic inflammation of the airway (50). Disruption of AJCs have been seen in response to RSV infection that led disruption of the barrier function of the epithelium predisposing to chronic inflammation (38). Some studies identified difference in the cytokine levels post viral infection in HNEC precipitating an inflammatory cascade e.g., IL 25 level escalation post influenza A infection (35), increased ephrin A1/A2 levels leading to type 2 inflammatory reaction (31), and elevated levels of CXCL-10 post RV infection (36). Although pretreatment with IFN-α was correlated with decrease in levels of inflammatory markers (35), Lee et al. found conflicting evidence as similar levels of antiviral IFN-*β* and IFN stimulated genes (ISGs) were found in subjects with CRSwNP and control group, post viral infection in cultured sinonasal epithelial cells, thus refuting their role in immune dysregulation seen in CRS patients (30).

In a mouse model that compared the rhinovirus infected area of nasal epithelium, cytokines and histology between a control group and induced chronic allergic rhinosinusitis group, there was a significant difference of concentration of viral particles per unit area of infected epithelium as measured by immunofluorescence in the study group and the levels of cytokines and the histology did not differ significantly between the groups (32).

In a study that compared atopic with non-atopic individuals, virus induced inflammatory response as measured by cytokine levels, differed in both groups during the acute as well as the convalescent phase. Thus, certain individuals may be more predisposed to developing inflammatory changes of the airway because of an acute viral infection (51). Interpersonal variation of inflammatory response to viral infection in CRS patients was also supported by the identification of rs6967330 risk allele associated with CDHR3 receptor gene, which is the receptor for RV-C and increased the odds of CRS in the patients harboring it (33).

**Figure 3:**
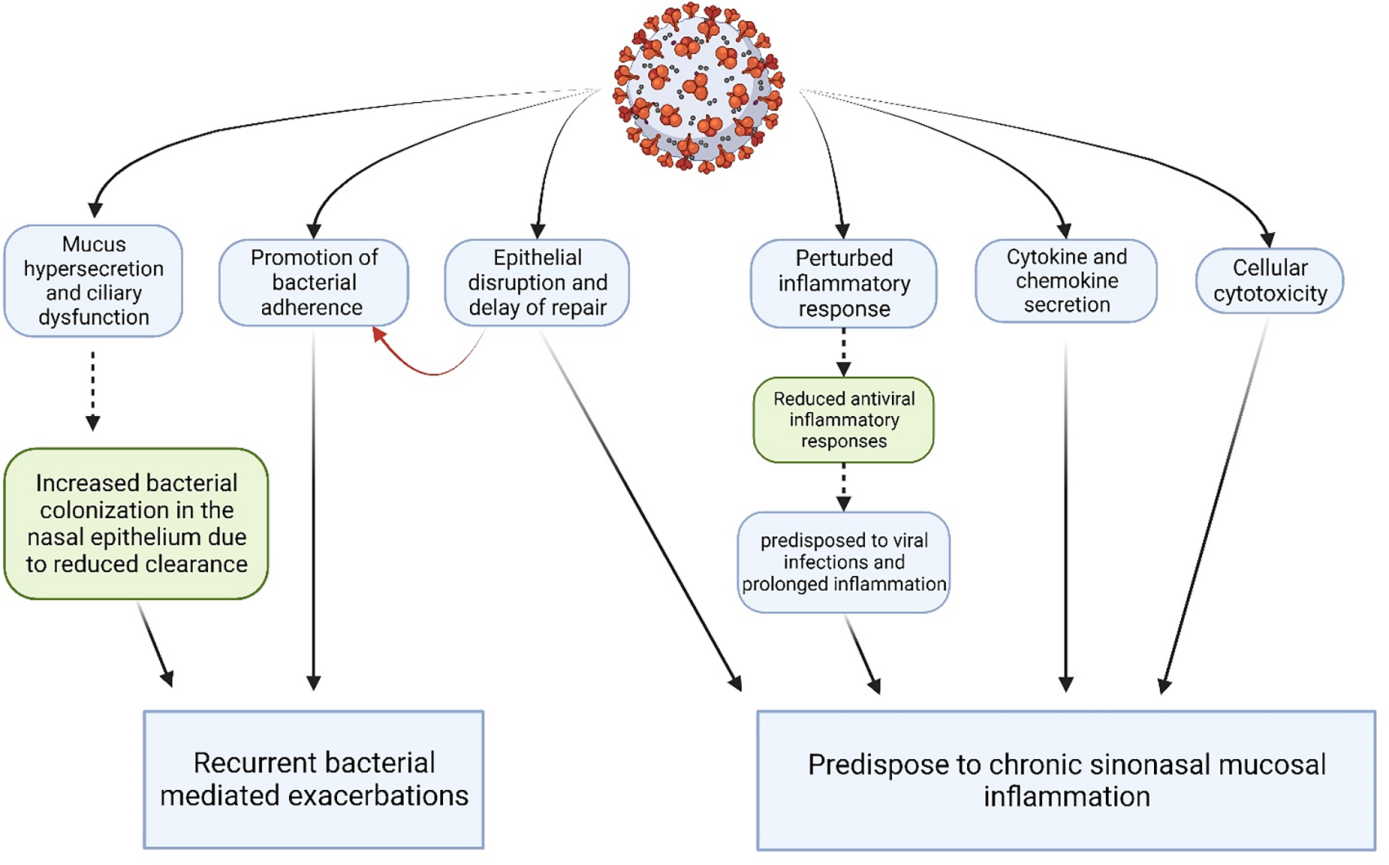
Synopsis of major pathogenic effects of viruses on the sinonasal epithelium. (Created with Biorender.com).

Figure 3 illustrates the various effects viruses may have on the airway epithelium. Several viruses that have been detected in patients of CRS are listed in Table 4 below.

**Table 4:** Viruses detected in patients of CRS.

Rhinovirus (12, 14, 15, 21, 26, 42) (most common according to Cho et al., Jang et al., Abshirini et al., Lima et al.)
Coronavirus (19, 21, 22) (most common according to Rowan et al.)
Influenza virus (15, 19, 21, 22)
Respiratory syncytial virus (12, 15, 19, 21, 22, 28)
Parainfluenza virus (15, 19, 22)
Adenovirus (12, 15, 21)
Enterovirus (15, 19, 21)
Bocavirus (15, 19, 21, 52)
Human metapneumovirus (most common according to Lima et al.) (15, 21)
Herpesvirus (23, 25, 29)

### 4.2. Possible role of viruses in CRS etiopathogenesis

Viruses may have a role in CRS disease initiation, exacerbation, and persistence.

#### 4.2.1. Initiation-trigger

Viruses may cause loss of epithelial integrity by viral induced cytotoxicity, epithelial barrier disruption, delayed and abnormal epithelial repair (38, 47, 53). Rezaee et al. demonstrated the early and late phase phosphorylation of PKD leading to cortactin phosphorylation destabilizing the actin cytoskeleton and disruption of AJCs like occludins, Zonula Occludens (ZO) −1 and tight junctions, confirmed by confocal microscopy and loss of TEES (transepithelial electrical resistance) in epithelial cell culture post infection with live RSV, and not with ultraviolet (UV) inactivated RSV (38).

Additionally, they may affect the immune system leading to the disruption of immune response towards the virus by subversion of IFN signaling (inhibition of IFN synthesis, inhibition of IFN downstream signaling) (18), expression of a Th2 dominated inflammatory pattern instead of Th1, and they may cause deleterious effects due to an exaggerated response (34, 47). Anti-viral IFN are the first line of defense against viral invasion in the sinonasal epithelium and have been hypothesized as the primary factor responsible for the dysregulated innate immune response leading to viral induction and exacerbation in CRS. Hong et al. confirmed the reduced viral invasion in IFN-α pre-treated airway epithelial cells (35), but Lee et al. found no difference in anti-viral IFN-β levels and ISGs between CRS patients' cultured sinonasal epithelial cells and control groups (30). In the search for factors responsible for immune dysregulation, ephrin A1/A2 was identified as phosphorylated (activated) and upregulated in viral infected CRS ethmoid mucosal cells; ephrin A1/A2 is speculated to increase levels of inflammatory mediators and downregulate the protective PI3K-AkT-NFκβ pathway which activates antiviral immune responses. Additionally, pretreatment with ephrin A1/A2 led to reduced antiviral IFN levels in the cell culture medium. These findings were absent when viral infected cells were treated with ephrin A2 blocker or ephrin A2 silencing RNA (siRNA), thus, confirming the role of ephrin A1/A2 in dysregulated immune response observed in CRS patients (31).

Viral infections also lead to increased mucus production, mucostasis and ciliary impairment (47, 50). They may promote bacterial infection through synergy between respiratory viruses and bacteria, decreased clearance, facilitated bacterial penetration and viral induced bacterial adherence (39, 47). Wang et al. showed in an *in vitro* study using 7 CRS patients and 10 controls that HSV-1 may facilitate invasion of *S. aureus* into the nasal mucosa (39). In another study using RV-16, Wang et al. demonstrated increased expression of CAMs like Fn (fibronectin), platelet activated factor (PAF) -γ, CEACAM (carcinoembryonic antigen associated CAM) determined by messenger RNA (mRNA) levels of their respective genes and via confocal microscopy. This was followed by increased adherence of bacteria like *S. aureus, S. pneumoniae, H. influenzae* to the HNEC (40). This study provides insights into how viral infections may predispose to secondary bacterial infections, as well as alter the microbiome which could be an important pathological mechanism behind periodic exacerbations of CRS.

While viruses may provide the trigger, disease initiation also depends on the host response. Proud et al. demonstrated in an *in vivo* study with experimental rhinovirus infection that expression of many genes including those associated with immune response, chemokines and antivirals is significantly altered (43, 48, 54). Identification of the rs6967330 genetic single nucleotide polymorphism (SNP) associated with the CDHR3 gene, which is a receptor for RV-C also led to the hypothecation of its role in viral associated CRS pathogenesis. Chang et al. found that the odds of CRS increased significantly when RV-C infection was found along with CDHR3 gene mutation, irrespective of asthma status (33). These groups of genes are likely to be the host factors in the virus associated pathogenesis of rhinosinusitis.

In a mice model, authors showed persistent hyperresponsiveness in nasal mucosa for at least 38 days, even after clearance of acute viral infection (42). This may suggest that viruses may provide an initial trigger that leads to an altered inflammatory response.

#### 4.2.2. Persistence and ongoing stimulus of chronic inflammation

##### 4.2.2.1. Data refuting role of virus in CRS persistence

There is a seasonal variation in the detection of respiratory viruses in the airway (55), with viral infections being more common in the winter months. Lima et al. analyzed this variation in CRS patients undergoing surgery and found that the same seasonal variation existed. The most frequent viruses detected by real time PCR (rt-PCR) were HRV and HMPV, and were the only viruses maintaining seasonal variation despite the detection during several periods of study. They studied this in nasal washes and sinus mucosa of 100 patients with CRS and thus, observed that this is a consequence of probable asymptomatic infection, and not of persistence of viruses in these patients, as the seasonal detection of viruses in CRS patients correlated with seasonal pattern of detection in other acute rhinitis patients. However, no controls were used in this study (21). In a study by Wood et al, tissue samples from 15 patients undergoing sinonasal surgery were analyzed by PCR assays for the presence of common respiratory viruses. All samples were negative for viruses. Out of these 15 patients, 2 were controls, 8 had CRSsNP and 5 had CRSwNP. They did further assays to look for Human herpes virus-6 (HHV-6) and Epstein Barr virus (EBV). Low titer HHV-6 was found in 3 of 8 CRSsNP, 4 of 5 CRSwNP and 1 of 2 normal subjects. Low titer EBV was found in 1 of 8 CRSsNP, 4 of 5 CRSwNP and 0 of 2 normal subjects. They concluded that persistence of respiratory viruses is not responsible for CRS. However, they collected the specimens only during the summer months, and the sample size was small (24). Liao et al. looked for the presence of viruses in the nasal mucosa from the middle meatus of 67 patients with CRSwNP, 61 CRSsNP and 53 controls. The viruses were detected using rt-PCR and the samples were equally distributed throughout the study period of almost 3 years. None of them had acute respiratory tract symptoms in the 4 weeks prior. They studied nine common respiratory viruses and did not find any significant difference in the overall viral detection rates, or individual viruses, across the three study groups. Also, they did not find any difference in disease severity within patients of CRS with and without viral detection. While their study spanned across all seasons, they suggest that they did not quantify the viral copies between the groups, which may be the cause of not finding a significant difference (22).

##### 4.2.2.2. Data supporting role of virus in CRS persistence

In an ex-vivo study that compared tissue from nasal polyps of patients with CRSwNP vs. inferior turbinate from healthy controls, a significant difference was seen in cytokine response on infection with HSV1. CRSwNP showed significantly higher IL-1β, IL-10 and TNF (tumor necrosis factor)-α, and significantly lower IFN-γ and IL-17 response (34). This deficient antiviral response as demonstrated by lower IFN-γ may be responsible for persistence of viruses in CRSwNP as compared to controls. Hwang et al. did not find a significantly different virus detection rate in mucosal scrapings from middle meatus in 35 eosinophilic CRSwNP, 38 non-eosinophilic CRSwNP, 36 CRSsNP and 21 controls but they also showed decreased levels of IFNs and IFN stimulated genes (ISGs) in patients of CRS, possibly showing an impaired antiviral response (18). In a study by Cho et al, the presence of respiratory viruses in 111 patients of CRS and 50 controls was studied using the PCR technique using two methods—nasal lavage samples and scrapings from inferior turbinate. A significant difference was seen in the overall viral detection rate and in the detection of Rhinovirus in CRS vs. controls. For parainfluenza, influenza, and RSV though there was a higher detection rate in CRS vs. controls, but it was not statistically significant. Overall, 64% of CRS samples detected positive for the presence of a respiratory virus. These patients had been selected after excluding those that had acute viral upper respiratory tract symptoms in the 4 weeks prior. Therefore, the presence of viruses, in the absence of symptoms may be due to asymptomatic infection, the incubation period before the onset of symptoms, or the persistence of viruses in CRS patients. However, they did not study the sinus mucosa (15). Jang et al. conducted a study on 39 patients with CRS and 29 controls who did not have any acute upper respiratory tract symptoms in the prior 4 weeks. 21 percent of CRS patients were positive for rhinovirus, and none of the controls were positive. They used nasal lavage samples and inferior turbinate scrapings. Whether the presence of virus was due to a new subclinical infection due to increased susceptibility to rhinovirus infections, or persistence from a previous infection, is difficult to establish (14). They did not study the sinus mucosa, however, it's persistence in the absence of acute symptoms of upper respiratory tract infection (URI) may suggest possibilities like in asthma, where rhinovirus RNA has been found in 32.4% of children with asthma, and is a possible important factor in the pathogenesis of the disease (56). Lee et al. to attempted determine HRV serotypes in 111 CRS patients and 51 non-CRS controls. No participant had an upper respiratory tract infection in the prior 4 weeks. HRV was detected using PCR technique in 40 CRS subjects (36%) and 11 non-CRS controls (21%). The overall detection rates of HRV in CRS patients from nasal lavage fluid and inferior turbinate epithelial cells were significantly higher than in non-CRS controls (13). Abshirini et al. compared for the presence of rhinovirus and RSV in the sinus mucosa of patients undergoing functional endoscopic sinus surgery (FESS). They found that the prevalence of at least one virus was 32.89% (26). Cho et al. detected a higher rate of viruses, probably also because they studied for the presence of 11 viruses, as compared to 2 viruses by Abshirini et al. (15, 26).

In another study, Goggin et al. investigated on 24 patients with CRS, and established a discord between the middle meatus and inferior meatus with respect to virus sampling (20). This may be an important factor in different studies leading to such varying results.

Goggin et al. next published their study of 288 patients, with 71 controls, 133 subjects with CRSsNP, and 84 CRSwNP. They used cytobrush samples from sinus mucosa and performed PCR for detection of common respiratory viruses. RV and coronavirus were the most common detected viruses. The maximum association with viral detection was found with CRSsNP and only CRSsNP subjects showed worsened sino-nasal outcome questionnaire test (SNOT) 22, endoscopic and radiological report, correlating with viral detection (19). Zaravinos et al. looked for the presence of HHV, HPV, EBV and CMV in 23 CRSwNP vs. 13 controls and found that statistically significant higher levels of EBV were found in CRSwNP. They sampled the polyp tissue and nasal mucosa from middle and inferior turbinates (25). Costa et al. in a case-only study on CRSwNP found that 60% of patients were positive to at least one virus, and EBV was seen in higher frequency in polyps and HHV-6 in healthy turbinate mucosa, though no statistically significant association was seen (23). Tao et al. also found that 85% of the 13 CRSwNP patients tested positive for EBV in polyp tissue, though very low numbers of EBV positive cells were found in each case, implying a possible role of viral persistence (29). However, these both studies were without controls. In a study that looked at differences in sero-reactivity between CRS and controls, Lal et al. found significantly elevated sero-reactivity in CRS patients against HMPV, HHV-4 and HHV-5 (17). In another case-only study to detect for the presence of adenovirus and RSV in 20 CRS patients undergoing surgery, 20% of the cases were positive for RSV, and none for adenovirus (28).

In summary, the association with viruses has been limited by incidental detection of virus in patient with CRS vs. controls. Only a few studies investigated viral detection with correlating measures of symptoms, radiological, endoscopic findings, or with inflammatory markers.

#### 4.2.3. Exacerbation

Demonstration of increased bacterial adherence following viral infection by HSV-1 (39) and RV (40) may indicate a mechanism of periodic exacerbation in CRS involving bacterial mediated inflammatory response. While the role of virus in exacerbation has been speculated, ascertaining their role has been challenging.

In a longitudinal study by Hardjojo et al., infants (205 samples from 32 subjects with prolonged/ recurrent rhinitis and 215 samples from 32 matched controls—healthy infants) were followed up to 18 months of age and presence of virus in anterior nasal swab was detected by PCR at regular quarterly visits, which revealed that presence of virus in the swabs obtained in 1 month pre and post rhinitis period was significantly higher in recurrent rhinitis group, indicating the possible role of viruses in periodic exacerbations (57).

Tacon et al., in an *in vitro* and *in vivo* study demonstrated that the destruction of the epithelial layer and macrophage recruitment due to RV infection induces production of MMP (matrix metalloproteinase) −9 (58). Wang et al. performed an *in vitro* study and found that rhinoviral infection enhanced the gene and protein expressions of MMP-2, MMP-9, and VEGF in nasal polyp fibroblasts, derived from polypoidal nasal tissue, implying the possible role of viruses in exacerbation of CRSwNP (41). Matrix metalloproteinases (MMPs) comprise a family of Ca2+-activated, Zn2+-dependent endopeptidases that participate in the degradation of extracellular matrix (ECM) (59). Vascular endothelial growth factor (VEGF) induces endothelial cell proliferation and vascular hyperpermeability (60). Both have been suspected to play an important role in pathogenesis of nasal polyposis.

Oncostatin M (OSM), a cytokine belonging to IL-6 family, is found to increase significantly following influenza A infection. In the study by Tian et al., OSM levels in nasal polyps of CRSwNP vs. inferior turbinate tissue from controls were found to be higher in the former, by analysis of the mRNA levels of OSM gene. Higher levels of OSM have been associated with epithelial barrier dysfunction. Their findings suggested that OSM could be expressed by both ciliated and goblet cells, disrupting the tight junctions following viral infections, and possibly exposing the subepithelia to invading pathogens to elicit inflammatory responses, causing exacerbations in CRSwNP (37). Alho et al. looked for the presence of virus during a “natural cold” in those with recurrent sinusitis vs. healthy controls. They used nasal mucosal biopsies with viral culture, antigen detection and PCR methods to maximize viral detection rates. They found 14 of 19 recurrent sinusitis patients detected positive for a respiratory virus and 12 of 20 healthy controls were positive. No significant difference could be found denying the viral associated exacerbation of rhinitis (45).

Rowan et al. used brush swabs from middle meatus or ethmoid sinus to collect samples from 13 CRSwNP, 8 CRSsNP and 14 controls to detect a panel of respiratory viruses and found viral presence more commonly in CRS patients. They further used sinonasal questionnaire, modified Lund-Mackay and modified Lund-Kennedy scores to determine the severity of symptoms in these patients. The results indicated predominant association of viruses with CRSsNP group (50% incidence) and only 8% in CRSwNP group; and non-significant symptomatic score, radiographic, and endoscopic differences between viral and non-viral associated CRS. Hence, they were unable to display an exacerbated symptomatology in viral associated CRS patients, although establishing their association with CRSsNP (12).

Divekar et al. conducted a prospective study in which CRSwNP cases and controls were asked to self-report immediately during exacerbation. Viral detection was done to find any difference in viral association in exacerbation of cases and controls. No significant difference in RV detection rates using PCR on nasal secretions was found during acute exacerbations in 9 CRSwNP vs. 10 controls (27).

In summary, study of viral association in CRS exacerbations was attempted using immunological markers or identification of virus itself. Of the 6 studies referenced, 3 supported the association whereas 3 couldn't identify viruses with CRS exacerbations.

## 5. Conclusions

A systematic review of the published data provides insufficient evidence regarding the conclusive role of viruses in CRS pathogenesis and exacerbations. Evidence suggests some probable higher prevalence of virus in the CRS subjects. CRS hosts may also possess immune characteristics that make them susceptible to virus infection and vulnerable to persistent sinonasal infections. Further studies on causation vs. association, possible mechanisms like subsequent immune dysregulation and epithelial instability with viral infections are necessary to provide further clarity to results from the current literature. To comprehensively evaluate the role of viruses in CRS with certainty, large studies with longitudinal sampling at all disease phases (i.e., prior to disease initiation, during disease initiation, during disease persistence, and during exacerbations) using standardized sampling techniques may be required. While such studies may be expensive to conduct, ascertaining the role of viruses may have important implications in the treatment and prevention of chronic rhinosinusitis.

## Data Availability

The original contributions presented in the study are included in the article/Supplementary Material, further inquiries can be directed to the corresponding author.
